# Pore Geometry and Nitrogen Doping Regulate Carbon-Source Transport Through Carbon Nanocage Shells for CO_2_ Electroreduction

**DOI:** 10.3390/nano16161002

**Published:** 2026-08-14

**Authors:** Cao Zhou, Zehan Yu, Lijun Yang

**Affiliations:** State Key Laboratory of Coordination Chemistry and Key Laboratory of Mesoscopic Chemistry of MOE, School of Chemistry and Chemical Engineering, Nanjing University, Nanjing 210023, China; all7n_cresearch@163.com (C.Z.); wo22shiniyeye@163.com (Z.Y.)

**Keywords:** CO_2_ electroreduction, N-doped graphene, micropores, molecular dynamics, interfacial transport

## Abstract

Carbon nanocages provide confined reaction environments for CO_2_ reduction reaction (CO_2_RR), but carbon sources must first cross their microporous graphitic shells to reach encapsulated catalytic sites. Here, molecular dynamics simulations were used to elucidate carbon-source transport through through-layer pores (TLPs), representing the edge-rich vertical micropores formed across stacked graphene layers in carbon nanocages. We examined the effects of pore diameter, N-doping, and pore depth on the transport of CO_2_RR-relevant carbon species. Among the investigated structures, a 12.1 Å N-doped TLP achieved the highest area-normalized cross-pore transport ratio of 1.296 × 10^−2^ Å^−2^. N-doping preferentially enhanced neutral CO_2_ transport, while producing only limited improvements for bicarbonate and carbonate ions. This selectivity originates from strong CO_2_ interactions with N-containing pore-edge sites, which establish a CO_2_-enriched interfacial region and promote adsorption-assisted capture–transfer without persistent molecular trapping. N-doping reduces both resistance contributions, leading to an approximately 30.4% decrease in pore-mouth resistance and ~45% reduction in pore-interior resistance at pore depths of 17.0 Å. These results identify short, appropriately sized, and N-functionalized through-layer micropores as favorable architectures for delivering CO_2_ to confined catalysts, providing molecular design principles for carbon-nanocage nanoreactors for CO_2_RR.

## 1. Introduction

Electrochemical CO_2_ reduction reaction (CO_2_RR) offers a promising route for converting carbon dioxide into value-added chemicals and fuels [1,2,3]. Beyond the intrinsic activity and selectivity of catalytic sites, the effective delivery of carbon-containing reactants to these sites is crucial, particularly under high-rate CO_2_RR conditions [4]. Recent comprehensive reviews have systematically emphasized that beyond the intrinsic activity and selectivity of catalytic sites, the effective delivery and mass transport of carbon-containing reactants (e.g., CO_2_ gas and dissolved anions) to these sites is crucial, particularly under high-rate CO_2_RR conditions [2,4,5]. This issue becomes especially important for confined catalysts, where active species are located inside nanoscale cavities and reactants must first traverse the enclosing shell before participating in interfacial reactions [6,7].

Hierarchical carbon nanocages provide an attractive platform for constructing such confined CO_2_RR catalysts [8]. Their distinctive architecture consists of nanosized hollow cavities surrounded by carbon shells containing subnanometer micropores. The cavity can accommodate and stabilize catalytic nanoparticles, while the micropores in the cage wall connect the internal confined space with the external electrolyte. Therefore, these micropores do not merely contribute to the surface area of carbon nanocages; they act as essential transport gateways that regulate the entry of carbon sources into the cage and their subsequent availability at encapsulated catalytic sites [9]. The size, chemistry, and local interfacial environment of these pores are thus expected to exert a decisive influence on CO_2_ transport [10]. Recent studies have shown that molecular transport through graphene-based nanopores is strongly regulated by pore-edge chemistry, dynamic pore structure, and the local molecular environment. In particular, pore-edge termination and dynamic structural fluctuations can substantially affect CO_2_ permeation and selectivity, while multilayer graphene pores and competitive CO_2_/H_2_O adsorption introduce additional transport constraints [11,12,13,14]. These studies provide relevant physical and methodological background for understanding carbon-source transport through graphitic nanopores.

Recently, we demonstrated that nickel nanoparticles confined within hierarchical N-doped carbon nanocages exhibit outstanding CO_2_-to-CO electroreduction performance [15]. In this confined configuration, however, the carbon source must overcome a multistep transport process: migration from the bulk electrolyte to the outer cage surface, capture at the pore mouth, passage through the microporous cage wall, and delivery into the internal cavity where the Ni-based active sites reside. Although the nanocage architecture provides confinement and structural stabilization, the cage wall may also introduce a mass-transfer barrier. Achieving efficient confined CO_2_RR therefore requires a balance between molecular accessibility through the shell and effective retention of carbon-containing species near the internal catalyst [16,17,18].

N-doping is widely employed to tune the electronic structure and surface chemistry of carbon nanocages [19,20,21,22,23]. In addition to modifying catalytic interfaces, N- functionalities may alter the interaction between carbon-source molecules and micropore walls, thereby changing molecular adsorption, diffusion, and trans-pore transport [24]. Nevertheless, how N-doping regulates carbon-source delivery through the cage wall, and how pore geometry and chemical interactions jointly determine transport resistance in confined carbon nanocage catalysts, remain poorly understood [16]. A molecular-level understanding of these processes is needed to establish rational design principles for nanocage-confined CO_2_RR catalysts.

Herein, molecular dynamics simulations were employed to investigate carbon-source transport across microporous graphene nanocages with different pore diameters, channel depths, and N-doped configurations. We reveal that carbon-source transport is governed by the competition between pore accessibility and interfacial capture, leading to an optimal pore diameter of 12.1 Å with the highest transport rate of 1.296 × 10^−2^ Å^−2^. More importantly, N-doping promotes carbon-source transport by strengthening favorable molecule–pore interactions and facilitating adsorption-assisted passage through the cage wall. By separating the total transport resistance into pore-mouth and intra-channel contributions, this work further clarifies the distinct roles of pore geometry and N-dopants in regulating molecular delivery to confined catalytic sites. These findings provide mechanistic guidance for designing N-doped carbon nanocages with efficient carbon-source transport for high-performance confined CO_2_RR catalysis.

## 2. Methods

All Molecular Dynamics (MD) simulations were performed using GROMACS (version 2019.6, University of Groningen, The Netherlands) [25]. The calculations aimed to investigate the transport kinetics at the electrolyte/electrode interface during the CO_2_RR process. To obtain sufficient statistics within a 10 ns time scale, a rigid graphene piston was introduced into the system, with the pulling reference coordinate set to advance along the Z-axis at a constant rate of 9.0 × 10^−4^ nm/ps to accelerate small molecules in the electrolyte and drive sustained convection–diffusion fluxes in the nanopores. Therefore, the obtained transport descriptors should be interpreted as comparative quantities under identical non-equilibrium driving conditions rather than as direct experimental fluxes. To verify the robustness of the key conclusions to the driving speed, additional simulations were conducted on the 12.1 Å N-doped single-layer micropore at piston speeds of 3 × 10^−4^ and 6 × 10^−4^ nm/ps (Figure S5). The simulation box dimensions were 4.2 × 4.2 × 13.5 nm^3^, containing approximately 23,000 atoms (Figure S1a). Figure S1b displays the atomistic configurations of the four types of graphene micropores employed in this work (with diameters of 4.9, 7.3, 12.1, and 17.0 Å, respectively), where the pore channels penetrate through a single-layer graphene sheet. To construct the system while balancing experimental relevance and simulation statistical reliability, this study optimized the electrolyte component concentrations in the simulation box. Specifically, 10 CO_2_, 20 HCO_3_^−^, and 10 CO_3_^2−^ were added to the system, with corresponding numbers of OH^−^ anions and Na^+^ cations supplemented to maintain system electroneutrality. To shorten the sampling time, the ion concentrations were slightly higher than typical experimental values, thereby obtaining sufficient molecular collision events at moderate computational cost. Pure water was placed in the chamber on the other side of the micropore to generate a chemical potential difference. This pure-water chamber was used as a sink region to maintain a concentration gradient and mimic carbon-source delivery from the external electrolyte into an initially depleted confined cavity. Figure S1c presents the graphene piston displacement (left axis) and the number of trapped particles between the piston and the pore mouth (right axis) as functions of simulation time. The piston displacement increases approximately linearly to 49.9 Å within 10 ns, ensuring a constant driving force for steady-state mass transport. The number of trapped particles rises briefly during the first 2 ns and then rapidly decreases to approximately 9800, indicating that the initial stage primarily involves the compression and rearrangement of molecules within the chamber; thereafter, the system enters a steady-state transport regime, where all species achieve stable cross-pore transport. The above results provide a reliable time window for the calculation of all subsequent transport parameters. Each configuration was independently simulated multiple times, and the resulting trajectories were averaged to suppress statistical noise and ensure the reproducibility of transport parameters.

This study employed the all-atom OPLS-AA force field to describe intermolecular interactions [26], with the SPC/E model used for water [27]. The force field parameters for electrolyte molecules were taken from references [28,29,30,31]. The micropore edges were passivated with hydrogen or modified by incorporating pyridine N. Periodic boundary conditions were applied to the simulation box in all three dimensions. The system was first relaxed in the NPT ensemble using the Berendsen semi-isotropic pressure coupling scheme for 2 ns, with a reference pressure of 1 bar. Subsequently, equilibration was performed in the NVT ensemble using the Nosé-Hoover thermostat at 300 K, with a thermostat time constant of 0.5 ps, for 3 ns [32]. After equilibration, a 10 ns non-equilibrium NVT simulation was conducted, with an integration time step of 1 fs, saving a trajectory frame every 2 ps. Five independent simulations were performed for each pore size sample to improve statistical reliability. Nominal electrode-potential conditions were approximated by estimating the surface charge density using a parallel-plate capacitor model and uniformly distributing the corresponding net charge over the electrode carbon atoms. Long-range electrostatic interactions were handled using the particle mesh Ewald method, and van der Waals interactions were truncated at 14.5 Å. Trajectory analysis was completed using VMD (version 1.9.3, University of Illinois at Urbana-Champaign, USA) and OVITO (version 3.6.0, OVITO GmbH, Darmstadt, Germany) [33,34]. The cross-pore transport rate was defined as the number of molecules that successfully crossed the micropore divided by the initial number of the corresponding species in the feed chamber. For area-normalized comparison, this value was further divided by the pore area. Therefore, the area-normalized cross-pore transport rate has a unit of Å^−2^. This descriptor was used to compare transport efficiencies among different pore sizes and chemical structures under the same simulation protocol.

## 3. Results and Discussion

**Figure 1:**
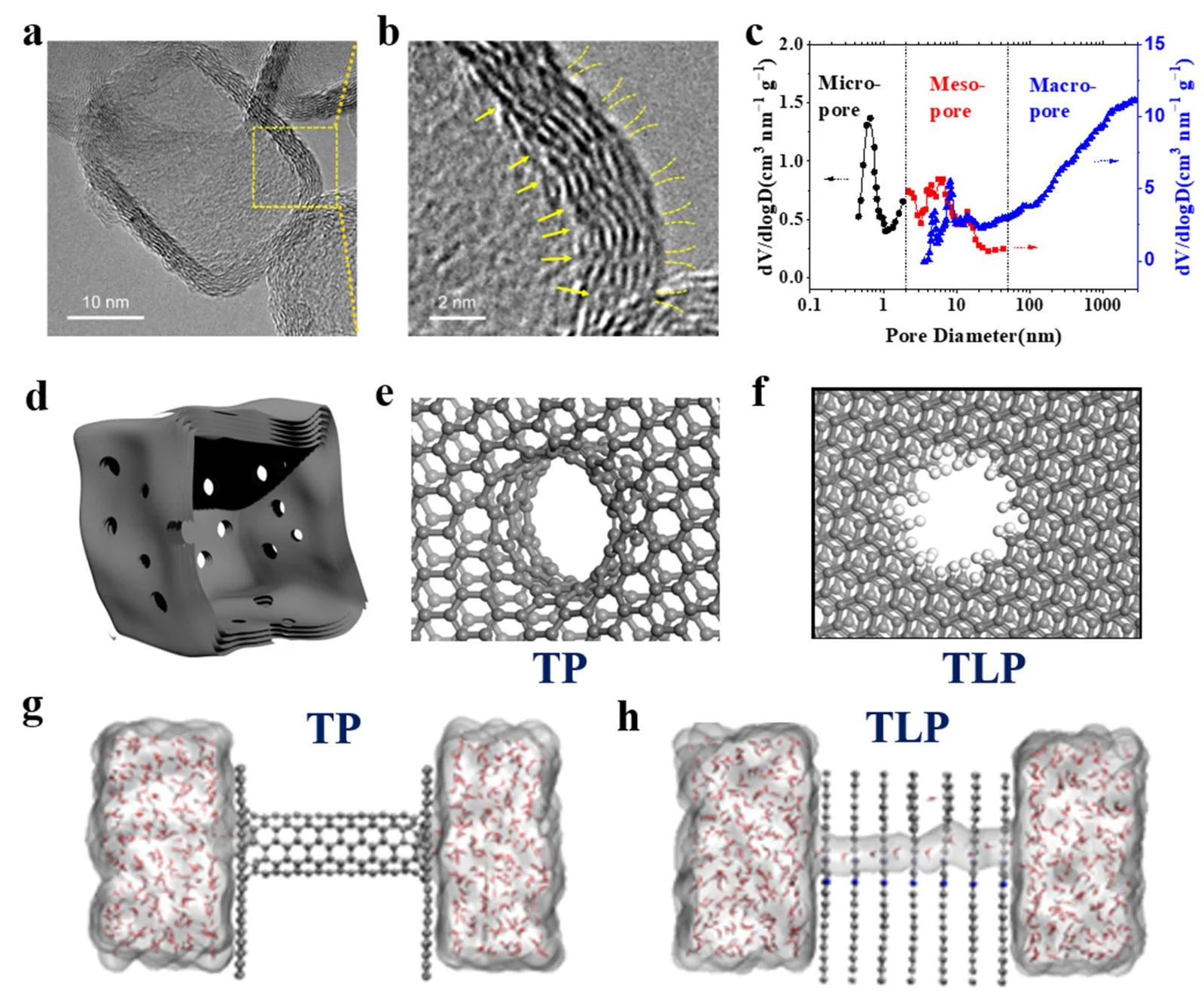
Identification of a representative micropore model for carbon nanocage shells. (**a**) Schematic illustration of a hierarchical carbon nanocage composed of stacked graphitic layers, highlighting micropores oriented normal to the carbon layers as transport gateways between the external electrolyte and the confined inner cavity. (**b**) Representative structural illustration of the graphitic shell and through-shell micropores. (The yellow arrow indicates the position of the micropores.) Reproduced with permission from Ref. [8]. Copyright 2026 American Chemical Society. (**c**) Representative pore-size distribution of carbon nanocages, where the regions corresponding to micropores, mesopores, and macropores are indicated. Reproduced with permission from Ref. [9]. Copyright 2015 Elsevier. (**d**) Schematic of a hierarchical carbon nanocage. (**e**,**f**) Atomic models of a tube pore (TP) and a through-layer pore (TLP) formed by aligned openings in stacked graphene layers. (**g**,**h**) Water-accessibility simulations for 7 Å TP and TLP. No water enters the TP within 10 ns, whereas the TLP forms a continuous water channel.

To establish a structurally relevant model for mass transport across carbon nanocage shells, we first focused on micropores oriented normal to the graphitic layers. Carbon nanocages consist of hollow shells assembled from stacked graphitic carbon layers, and their subnanometer micropores serve as the primary gateways connecting the external electrolyte with the internal cavity (Figure 1a–c). Unlike mesopores and macropores, which mainly facilitate bulk diffusion and electrolyte storage, these micropores directly determine whether carbon-source species can access catalysts confined inside the nanocage.

The atomic-scale structure of such through-shell micropores remains ambiguous. Considering the stacked graphitic architecture of carbon nanocages and the exposed edge sites along the pore wall, two representative pore configurations were considered (Figure 1d–f). The first is a tube pore (TP), represented by a smooth cylindrical channel analogous to a short carbon nanotube (Figure 1e). The second is a through-layer pore (TLP), constructed by aligning pores in stacked graphene layers along the direction normal to the carbon sheets (Figure 1f). In the TLP model, the pore wall is composed of graphene edges and therefore presents a chemically and geometrically heterogeneous environment, where edge functional groups may exist under realistic conditions. This structure is more consistent with a micropore formed across multilayer graphitic shells.

To distinguish which model better represents the transport behavior of carbon-nanocage micropores, we performed a water-permeation test using TP and TLP structures with the same pore diameter of 7 Å (Figure 1g,h). Water layers were placed on both sides of the pore, followed by 10 ns molecular dynamics simulations. As shown in Figure 1g, water molecules did not enter the TP during the entire simulation period, and no continuous water pathway was formed. In contrast, water readily penetrated the TLP and established a stable water channel across the multilayer pore (Figure 1h). This result demonstrates that the atomic structure of the pore wall significantly affects pore wetting and aqueous molecular accessibility. The smooth and highly confined TP structure introduces a substantial water-entry barrier, whereas the edge-rich TLP structure provides more favorable interactions and local configurations for water incorporation and trans-pore transport. Because water and CO_2_ have different molecular and solvation properties, the water-permeation result is used only as a structural screening criterion.

This distinction is particularly important for confined electrocatalysis in carbon nanocages. Previous studies of carbon-nanocage-confined catalysts for nitrate reduction have demonstrated that water molecules and ionic species can exchange between the inner cavity and the external electrolyte through shell micropores [35]. Such experimentally established species exchange requires the presence of water-accessible transport pathways. Therefore, a pore model that cannot support water penetration at a representative micropore size is unlikely to describe the operative transport channels in carbon nanocages. On this basis, the TLP model was selected as the primary structural model for the following simulations, which examine how pore size, pore depth, N-doping, and electrode potential regulate carbon-source transport toward catalysts confined inside carbon nanocages.

**Figure 2:**
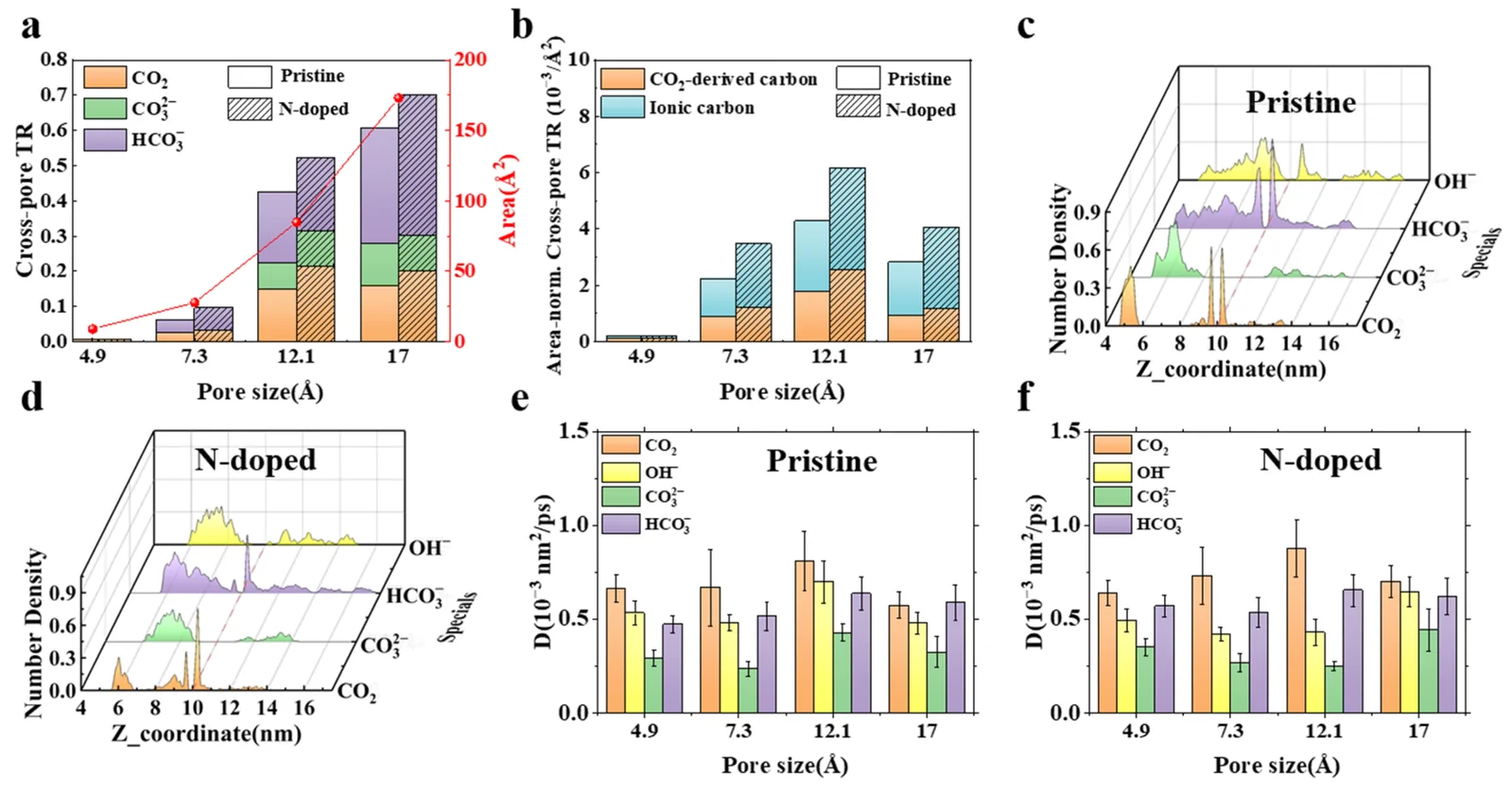
N-doping preferentially promotes CO_2_ transport through single-layer TLP. (**a**) Cross-pore transport ratios (TRs) of CO_2_RR-relevant carbon species and ionic species through pristine and N-doped single-layer TLPs with different pore diameters. (**b**) Area-normalized cross-pore transport rates (TRs) of CO_2_ and total carbon-containing species, showing the highest carbon-source transport efficiency for the 12.1 Å N-doped pore. (**c**,**d**) One-dimensional number-density profiles of CO_2_ along the transport direction for pristine and N-doped TLPs at 10ns, respectively. N-doping enhances CO_2_ accumulation near the pore interface and increases its downstream population. (**e**,**f**) Diffusion coefficients of CO_2_, HCO_3_^−^, and OH^−^ near pristine and N-doped pore interfaces, respectively, as a function of pore diameter. Error bars represent the standard deviation from five independent simulations.

Figure 2 summarizes the direct transport outcomes for CO_2_RR-relevant carbon sources and ions through single-layer TLPs, aiming to clarify (i) whether TLPs can serve as realistic exchange windows for confined nanocage catalysis, and (ii) how pore size and N-doping modulate the permeability and selectivity of these micropores.

As the pore diameter increases, the overall cross-pore transport rate (TR) rises (Figure 2a), consistent with a larger geometric opening providing a lower steric barrier and a wider passage. However, the response is not uniform across species. In particular, ionic species, especially CO_3_^2−^, remain strongly suppressed in the smallest pores, reflecting not only size exclusion but also the additional energetic penalty associated with hydration and partial desolvation when passing through subnanometer channels. These results support the view that TLPs can function as transport gateways, yet the effective “open” window for different carbon sources depends critically on pore size.

A key observation from Figure 2a is that N-doping increases cross-pore transport, but the magnitude of enhancement is species-dependent. For N-doped pores with diameters of 7.3, 12.1, and 17.0 Å, the CO_2_ TR increases by 33%, 49%, and 24%, respectively, relative to pristine pores. In contrast, the increases for carbon-containing ions (HCO_3_^−^, CO_3_^2−^) are comparatively modest. This indicates that N-doping primarily promotes the transport of the neutral molecular carbon source (CO_2_), rather than universally accelerating all charged carbon carriers.

This selectivity is important for confined CO_2_RR: if the catalyst is located inside the nanocage, efficient supply of CO_2_ to the interior is a direct prerequisite for sustaining high turnover, whereas ionic carbon species may be additionally constrained by electrostatic interactions and solvation barriers at the pore mouth and inside the channel.

To decouple pore-size effects from simply having more open area, we further compare area-normalized TR (Figure 2b). When CO_2_ is considered alone, the 12.1 Å N-doped pore shows the highest CO_2_ transport per unit area (5.15 × 10^−3^ Å^−2^), exceeding both smaller (7.3 Å) and larger (17.0 Å) pores. When all carbon-containing species are included, the total area-normalized carbon transport at 12.1 Å reaches 1.296 × 10^−2^ Å^−2^, which is 1.25× that of the pristine pore at the same diameter. These results together suggest that within the tested range, a pore size near 12.1 Å provides the most effective “carbon-supply window”, where the channel is sufficiently open to allow frequent crossing, while still offering interfacial interactions that favor successful translocation (particularly for CO_2_). The highest area-normalized transport at 12.1 Å reflects a balance between geometric accessibility and productive pore-mouth transport. Increasing the pore size from 12.1 to 17.0 Å increases the effective pore area by approximately 2.04-fold, whereas the CO_2_ transport ratio does not increase proportionally.

**Figure 3:**
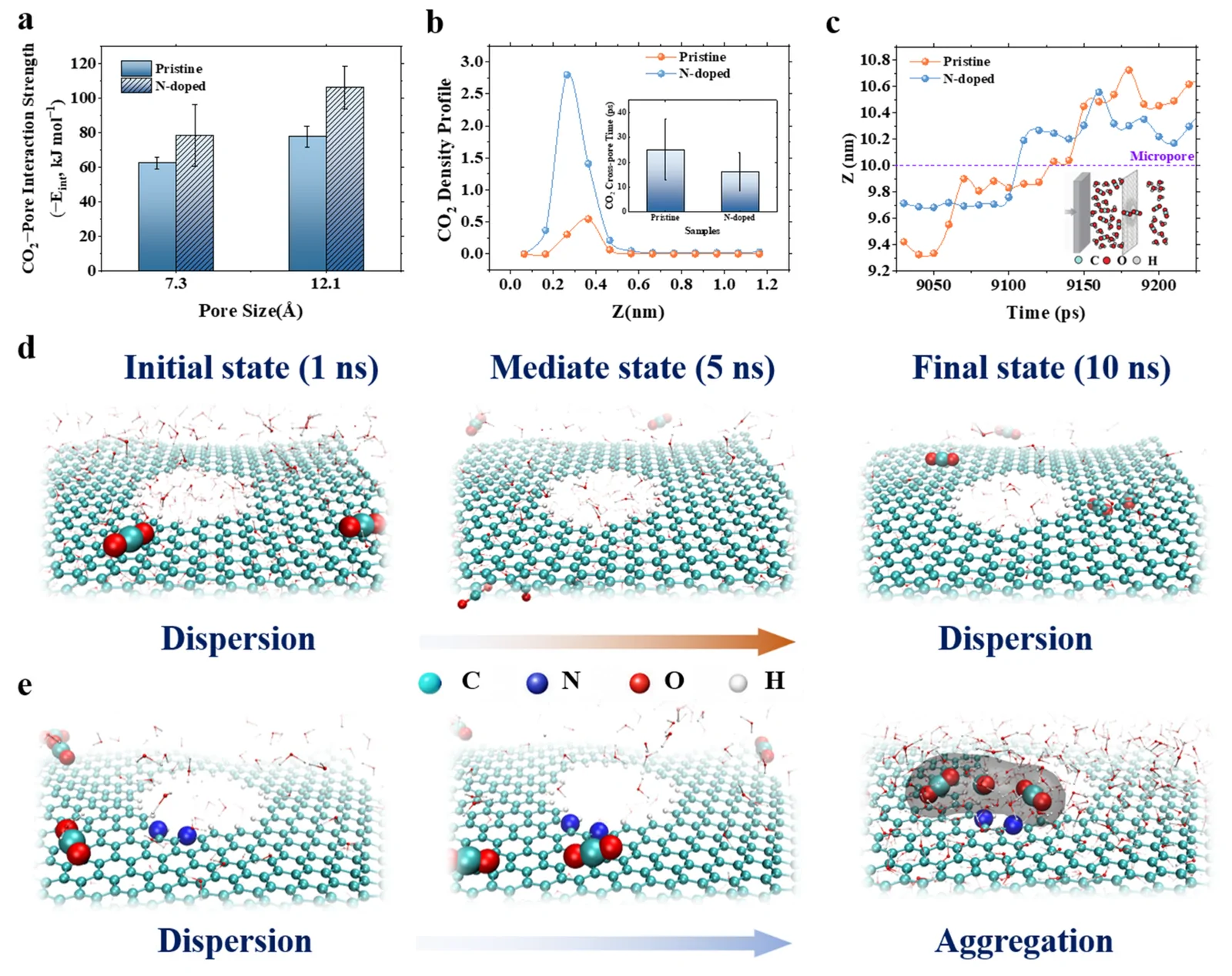
Molecular origin of N-doping-enhanced CO_2_ transport through single-layer TLPs. (**a**) Average interaction energies between CO_2_ and pristine or N-doped TLPs with pore diameters of 7.3 and 12.1 Å. (**b**) Normalized one-dimensional CO_2_ number-density profiles along the transport direction near pristine and N-doped 12.1 Å TLPs with time averaging. Inset: cross-pore times of CO_2_ molecules that successfully traverse the pore. (**c**) Representative trajectories of the CO_2_ carbon atom along the transport direction in the N-doped 12.1 Å TLP. Representative MD snapshots showing the time-dependent CO_2_ distribution near pristine (**d**) and N-doped (**e**) 12.1 Å TLPs.

The one-dimensional number-density profiles at 10 ns along the transport direction provide a qualitative view of interfacial species distributions (Figure 2c,d). In pristine pores, CO_2_ shows enhanced density adjacent to the carbon layers, consistent with interfacial adsorption. After N doping, the downstream CO_2_ density increases, consistent with the greater number of molecules that successfully traverse the pore. As these profiles cannot distinguish pore-mouth adsorption from adsorption on carbon surfaces away from the pore, the spatial localization and dynamics of CO_2_ near the pore are further examined in Figure 3. Ionic carbon species show only modest changes in their density profiles after N doping. Their transport remains more strongly constrained by hydration and electrostatic interactions, consistent with their smaller increase in transport ratios relative to neutral CO_2_.

The diffusion coefficients extracted near the pore mouth (Figure 2e,f) show that the mobility of several species (CO_2_, HCO_3_^−^, OH^−^) generally increases with pore size but may level off or slightly decrease at the largest diameter, suggesting that the transport process is governed by a balance between confinement, interfacial interactions, and local structuring of water/ions near the pore. N-doping further perturbs this interfacial environment, consistent with its strong impact on CO_2_ passage.

Finally, under applied potentials (Figure S2), we observe opposite trends for CO_2_ and HCO_3_^−^: more negative potentials suppress HCO_3_^−^ transport while increasing CO_2_ transport. This divergence reinforces that neutral and ionic carbon sources experience distinct interfacial gating mechanisms, and that optimizing carbon delivery into confined nanocages cannot be reduced to pore size alone.

To clarify why N-doping selectively enhances CO_2_ transport through TLPs, we compared the CO_2_–pore interactions in pristine and N-doped pores with diameters of 7.3 and 12.1 Å (Figure 3a and Figure S3a). At both pore sizes, N-doping substantially strengthened the attractive interaction between CO_2_ and the pore edge. For the 7.3 Å pore, the average CO_2_–pore interaction energy decreased from −62.5 kJ mol^−1^ for the pristine pore to −78.4 kJ mol^−1^ after N-doping. The effect was more pronounced for the 12.1 Å pore, where the interaction energy changed from −77.9 to −106.3 kJ mol^−1^. These results indicate that pyridinic N sites at the exposed graphene edges create a more favorable interfacial environment for CO_2_ capture.

The 12.1 Å pore was selected for mechanistic analysis because it delivered the highest area-normalized carbon-source transport. The one-dimensional CO_2_ number-density profiles show a pronounced enrichment near the entrance of the N-doped pore, whereas CO_2_ remains much more diffusely distributed around the pristine pore (Figure 3b). Thus, N-doping does not simply accelerate the passage of CO_2_ already located inside the pore. Rather, it increases the local CO_2_ availability at the pore mouth, thereby raising the probability that a CO_2_ molecule enters the transport pathway. To distinguish pore-mouth capture from productive translocation, we compared the reach-pore transport ratio with the cross-pore transport ratio. The former represents CO_2_ molecules reaching the pore-mouth region, whereas the latter represents molecules successfully traversing the pore. N-doping increases both ratios (Figure S6a), while the pore-mouth residence time of all resolved entry events remains comparable to or lower than the cross-pore times of successfully traversed CO_2_ molecules (Figure 3b and Figure S6b inset). These results support an adsorption-assisted capture–transfer mechanism without persistent kinetic trapping within the sampled trajectories.

Importantly, stronger CO_2_ accumulation does not lead to kinetic trapping. The successful crossing events exhibit a shorter cross-pore time in the N-doped system than in the pristine pore (Figure 3b, inset). The trajectory analysis further shows more rapid and continuous displacement of CO_2_ along the transport direction after N-doping (Figure 3c). Together, these results suggest a capture–transfer mechanism: N-containing edge sites first enrich CO_2_ at the pore–electrolyte interface and then facilitate its onward migration through the pore, rather than immobilizing it at the entrance.

The molecular trajectories provide a direct visualization of this process. In the pristine pore, CO_2_ molecules remain sparsely dispersed near the pore region throughout the simulation, leading to infrequent productive entry events (Figure 3d). By contrast, the N-doped pore establishes a persistent CO_2_-rich zone at the pore mouth, from which repeated trans-pore migration events occur (Figure 3e). N-doping therefore improves CO_2_ transport through a balanced interfacial function: it increases CO_2_ capture sufficiently to create a locally enriched reactant reservoir, while preserving rapid passage across the TLP.

These findings explain the preferential increase in CO_2_ transport observed in Figure 2. Unlike hydrated ionic carbon species, whose passage is constrained primarily by desolvation and electrostatic penalties, neutral CO_2_ can benefit directly from the strengthened yet non-trapping interaction with N-containing pore edges. The pore-edge chemistry of carbon nanocages can therefore regulate carbon supply to confined catalysts by selectively enriching and transmitting molecular CO_2_.

**Figure 4:**
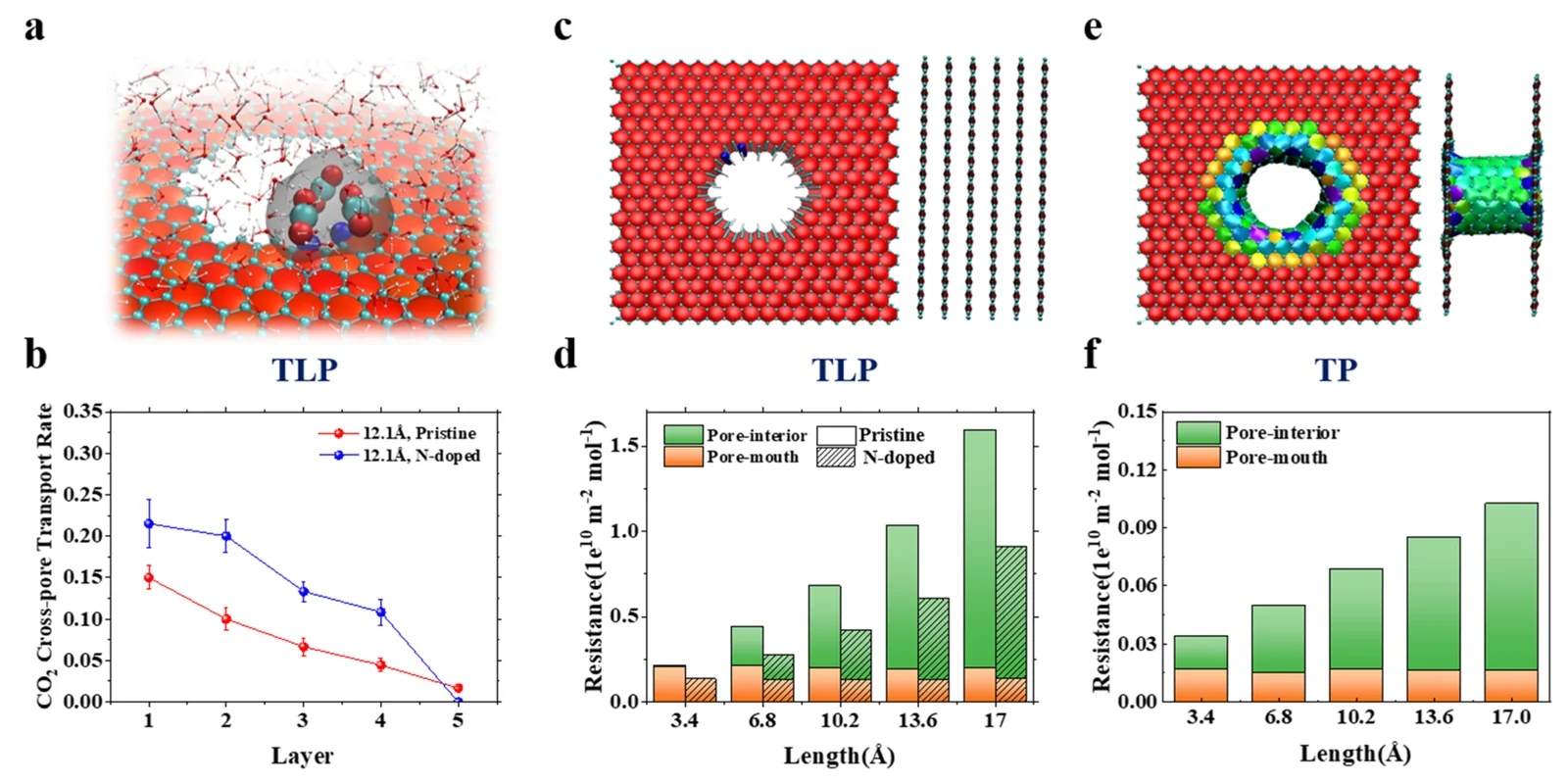
Pore depth governs CO_2_ transport resistance in TLPs, while tube-like channels provide a low-resistance structural reference. (**a**) Representative nonequilibrium MD snapshot of CO_2_ transport through TLPs. (**b**) CO_2_ transport rate through pristine and N-doped 12.1 Å TLPs as a function of pore depth, corresponding to one to five graphene layers. (**c**) Top and side views of the multilayer TLP model. (**d**) Decomposition of CO_2_ transport resistance in pristine and N-doped TLPs into pore-mouth and pore-interior contributions. (**e**) Top and side views of the TP reference model with the same nominal pore size and comparable channel depths. (**f**) Corresponding pore-mouth and pore-interior resistance contributions for TPs.

After establishing the single-layer TLP as the representative model for carbon-nanocage shell micropores, we next examined how shell thickness influences CO_2_ delivery to confined catalysts. Multilayer TLPs with a fixed pore size of 12.1 Å were constructed by stacking one to five graphene layers, corresponding to pore depths of 3.4–17.0 Å (Figure 4a–d). This analysis directly addresses a practical structural question for carbon nanocages: although a thicker graphitic shell may improve mechanical robustness, it also extends the confined transport path that carbon sources must traverse before reaching the inner catalyst.

The CO_2_ transport rate decreases monotonically as TLP depth increases for both pristine and N-doped pores (Figure 4b). N-doping enhances transport at every depth, confirming that the favorable CO_2_ capture identified for single-layer pores persists in multilayer structures. Nevertheless, the benefit becomes progressively less pronounced as the channel length increases. At shallow depth, N-doping can effectively enrich CO_2_ at the pore entrance and promote productive crossing. In deeper pores, however, the gain from interfacial capture is increasingly offset by resistance accumulated during transport through the confined channel. At a depth of 17.0 Å, CO_2_ transport is low in both systems, indicating that excessively thick graphitic shells can severely restrict carbon supply to the encapsulated catalyst.

To identify the origin of this depth dependence, the total transport resistance was separated into a pore-mouth contribution and a pore-interior contribution. In pristine TLPs, the pore-mouth resistance remains nearly unchanged over the investigated depth range, varying by less than 10% (Figure 4c,d). By contrast, pore-interior resistance rises sharply with increasing depth, changing from a minor contribution in shallow pores to the dominant resistance component in deep pores. The N-doped TLPs show the same overall trend. At a pore depth of 17.0 Å, N-doping reduces the pore-mouth resistance by approximately 30.4% and the pore-interior resistance by approximately 45%, relative to the pristine TLP. Thus, N-containing pore edges facilitate not only CO_2_ capture at the interface but also transport within the confined pore.

As a structural reference, we then evaluated TPs with the same pore size and comparable depths (Figure 4e,f). This comparison is not intended to replace TLPs as the structural model of the present carbon nanocages; rather, it illustrates how a different pore architecture could alleviate the transport limitation identified above. In contrast to TLPs, TPs form smooth cylindrical channels with a much lower pore-mouth resistance. Although their pore-interior resistance still increases with channel length—by approximately fivefold from 3.4 to 17.0 Å—the entrance contribution decreases from 49.9% to 16.1% of the total resistance as the tube length increases. The lower interfacial barrier of TPs reflects the absence of repeatedly exposed graphene-edge environments along the transport pathway.

The TP comparison therefore provides a design implication rather than a competing structural description of the carbon nanocage shell. While TLPs are appropriate for describing the present material, nanoreactors containing short, open, and tube-like transport channels may enable more facile carbon-source delivery, particularly when a thicker protective shell is necessary. Engineering carbon nanocages toward reduced pore tortuosity, fewer serial edge barriers, or deliberately integrated tube-like conduits may thus help preserve confinement while mitigating mass-transfer resistance.

## 4. Conclusions

This work establishes a molecular-level framework for carbon-source delivery across the microporous shells of carbon nanocages containing confined CO_2_RR catalysts. Using TLPs, which reproduce the edge-rich vertical micropores of stacked graphitic shells, we show that pore geometry, pore-edge chemistry, and shell thickness collectively determine the accessibility of the inner catalytic space. Among the examined pore sizes, the N-doped 12.1 Å TLP provides the most favorable carbon-supply performance, reaching the highest area-normalized total carbon-source transport rate of 1.296 × 10^−2^ Å^−2^. N-doping preferentially promotes the transport of neutral CO_2_, whereas the transport enhancement for bicarbonate and carbonate species is comparatively limited. This selectivity arises from strengthened CO_2_–pore interactions at N-containing graphene edges, which generate a CO_2_-enriched interfacial region near the pore mouth without imposing persistent trapping. N-doping therefore enables an adsorption-assisted capture–transfer process that increases the probability of productive CO_2_ translocation into the nanocage interior. In TLP systems, for a fixed pore size, the pore-mouth resistance remains nearly invariant with pore depth, whereas the pore-interior resistance accumulates with increasing depth, giving rise to a nonlinear escalation of the total transport resistance. N-doping reduces both resistance contributions, leading to an approximately 30.4% decrease in pore-mouth resistance and ~45% reduction in pore-interior resistance at pore depths of 17.0 Å. These results indicate that the short and moderately sized microporous structure with N-functionalization through layers is an ideal configuration for transporting CO_2_ to the confined catalyst, providing a molecular design principle for the selective reduction in CO_2_ in carbon nanocage nanoreactors.

## Data Availability

The original contributions presented in this study are included in the article/Supplementary Materials. Further inquiries can be directed to the corresponding author.

## Supplementary Materials

The following supporting information can be downloaded at https://www.mdpi.com/article/10.3390/nano16161002/s1. Figure S1. (a) 3D simulation snapshot of the non-equilibrium MD simulation system, showing the electrolyte feed chamber (left), micropore/channel region (center), and the absorption chamber (right). (b) Atomistic construction of graphene micropores with different pore geometries and sizes (4.9, 7.3, 12.1,17.0 Å). (c) Graphene piston displacement (left axis) and number of trapped particles between the piston and the pore mouth (right axis) as functions of simulation time. Table S1. Summary of pore size connecting cavities in confining structures for CO_2_RR. Figure S2. (a) Cross-pore transport rates of carbon-containing species (CO_2_, CO_3_^2−^, HCO_3_^−^) through 12.1 Å micropores under nominal electrode potentials of 0 V, −0.52 V, and −1.05 V for pristine (open) and N-doped (hatched) pores. (b) Distribution of the angle between the C–O(H) bond orientation of HCO_3_^−^ and the Z-axis. (c) Average charge distribution on the electrode surface. (d) Average number of hydrogen bonds during micropore passage at electrode potentials of −0.52 V and −1.05 V. Figure S3. Interaction energies of (a) CO_2_, (b) HCO_3_^−^, and (c) CO_3_^2−^ with the micropore. Figure S4. (a) Snapshot of hydrogen bonding when a bicarbonate ion crosses the micropore/electrolyte interface. (b) Minimum distance between the ion and the hydrogen-terminated micropore during the crossing process. (c) Distribution of the angle between the C–O(H) bond and the Z-axis as HCO_3_^−^ passes through the interface. (d) Average number of hydrogen bonds along the Z coordinate for a nitrogen-doped four-layer graphene micropore. The purple dashed line indicates the pore position. Figure S5. Cross-pore transport rates through the 12.1 Å N-doped micropore at different piston speeds under U = 0 V. Figure S6. Comparison of CO_2_ pore reaching and cross-pore transport. (a) Reach-pore transport ratio and Cross-pore transport ratio for pristine and N-doped TLPs. (b) Pore-mouth residence time of all resolved entry events (Reach-pore transport) and cross-pore times of successfully traversed CO_2_ molecules. Figure S7. Schematic diagram of the calculation scheme for effective pore area. References [15,18,36,37,38] are cited in the Supplementary Materials.
