# Supplementary material for: Pore Geometry and Nitrogen Doping Regulate Carbon-Source Transport Through Carbon Nanocage Shells for CO2 Electroreduction
Source: Nanomaterials (Basel). 2026 Aug 14;16(16):1002. doi: 10.3390/nano16161002 (PMC13516970; doi:10.3390/nano16161002)
Supplement: Supplementary file 1 [file nanomaterials-16-01002-s001.zip › nanomaterials-4476400-supplementary.pdf]

Pore Geometry and Nitrogen Doping Regulate Carbon-Source Transport through Carbon Nanocage Shells for CO_2 Electroreduction

Cao Zhou, Zehan Yu and Lijun Yang *

State Key Laboratory of Coordination Chemistry and Key Laboratory of Mesoscopic Chemistry of MOE, School of Chemistry and Chemical Engineering, Nanjing University, Nanjing 210023, China; all7n_cresearch@163.com (C.Z.); wo22shiniyeye@163.com (Z.Y.)

* Correspondence: lijunyang@nju.edu.cn

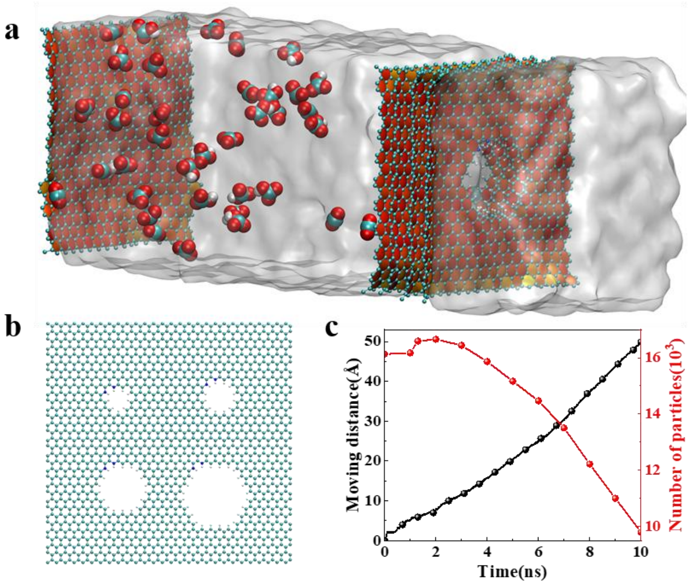

Figure S1. (a) 3D simulation snapshot of the non-equilibrium MD simulation system, showing the electrolyte feed chamber (left), micropore/channel region (center), and the absorption chamber (right). (b) Atomistic construction of graphene micropores with different pore geometries and sizes (4.9, 7.3, 12.1,17.0 Å). (c) Graphene piston displacement (left axis) and number of trapped particles between the piston and the pore mouth (right axis) as functions of simulation time.

Table S1. Summary of pore size connecting cavities in confining structures for CO_2RR.

Materials	pore size	Performance (vs RHE)	Ref
PdAg@NMHCS	0.6 nm	TOF_{2h}=340h^{-1}	[1]
Ni@hNCNC	0.62 nm	352 mA cm^{-2}(-1.1 V) (Alkaline)	[2]
Ni_5@NCN	2.06 nm	17.1 mA cm^{-2}(-0.8 V) (Neutral)	[3]
Ni@N-C	1.25 nm	236.7 mA cm^{-2}(-1.1 V) (Alkaline)	[4]
Ni-N/C-H-Meso	2.7 nm	16.2 mA cm^{-2}(-1.1 V) (Neutral)	[5]

The pore sizes and key structural parameters of experimentally reported CO_2RR nanocage materials are summarized in **Table S1**.

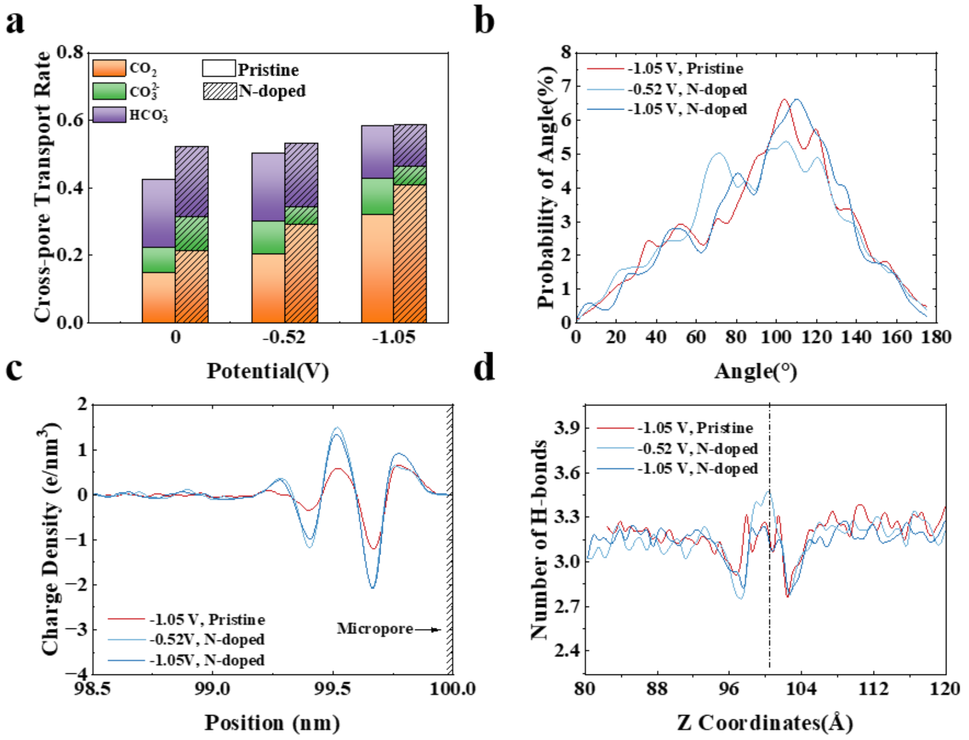

Figure S2. (a) Cross-pore transport rates of carbon-containing species (CO_2, CO_3^{2-}, HCO_3^-) through 12.1 Å micropores under nominal electrode potentials of 0 V, −0.52 V, and −1.05 V for pristine (open) and N-doped (hatched) pores. (b) Distribution of the angle between the C–O(H) bond orientation of HCO_3^- and the Z-axis. (c) Average charge distribution on the electrode surface. (d) Average number of hydrogen bonds during micropore passage at electrode potentials of -0.52 V and -1.05 V.

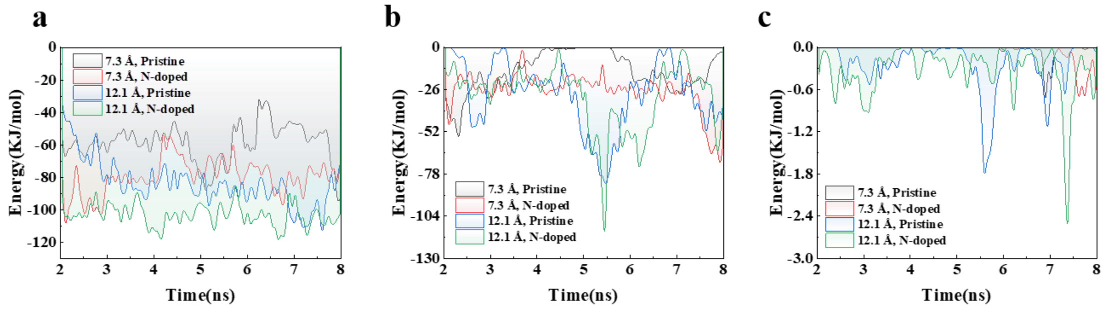

Figure S3. Interaction energies of (a) CO_2, (b) HCO_3^-, and (c) CO_3^{2-} with the micropore.

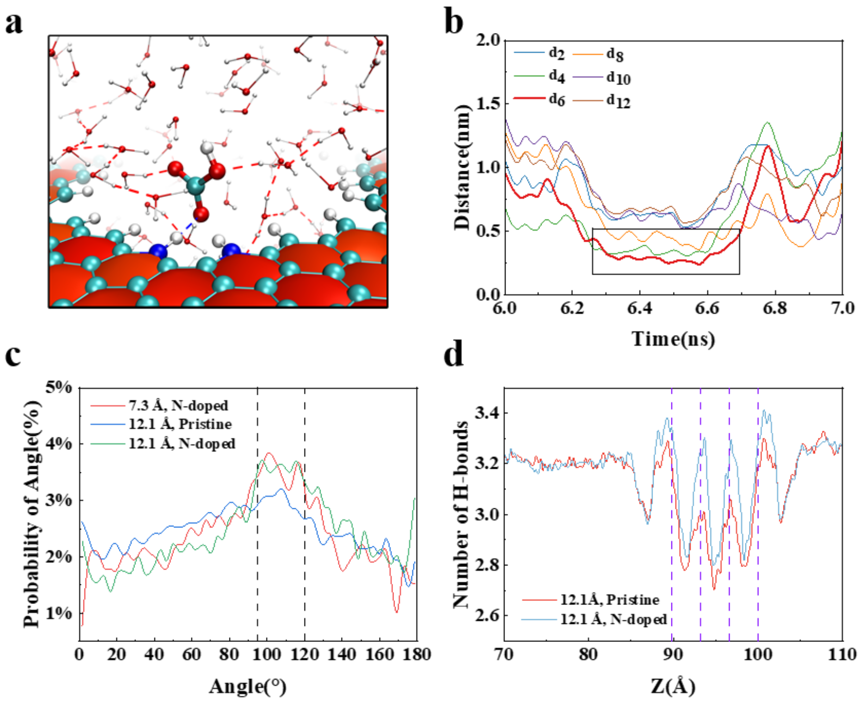

Figure S4. (a) Snapshot of hydrogen bonding when a bicarbonate ion crosses the micropore/electrolyte interface. (b) Minimum distance between the ion and the hydrogen-terminated micropore during the crossing process. (c) Distribution of the angle between the C–O(H) bond and the Z-axis as HCO_3^- passes through the interface. (d) Average number of hydrogen bonds along the Z coordinate for a nitrogen-doped four-layer graphene micropore. The purple dashed line indicates the pore position.

Figure S4 illustrates the molecular behavior of HCO_3^- during micropore/electrolyte interfacial traversal. **Figure S4a,b** show that when HCO_3^- approaches the doped pore mouth with an oxygen-end-down orientation, hydrogen bonding with hydrogen atoms near doping sites is activated, leading to short-term capture of the ion near the pore mouth (minimum distance of ~0.25 nm). Within ~0.1 ns, this distance rapidly rebounds to ~1.3 nm, indicating a rapid release-cross-pore event. Thus, HCO_3^- crossing of the micropore/electrolyte interface is characterized by short-time-scale capture-release dynamics driven by the interfacial hydrogen bond network, rather than a continuous single-step process. **Figure S4c,d** further reveal from a structural perspective that N-doped pores induce a preferred "flat-lying" orientation (θ in 95°–120°) and a more continuous hydrogen bond network in the channel interior (average hydrogen bond number increasing from 2.75 to 3.2), providing a locally favorable interfacial environment for bicarbonate near the pore mouth.

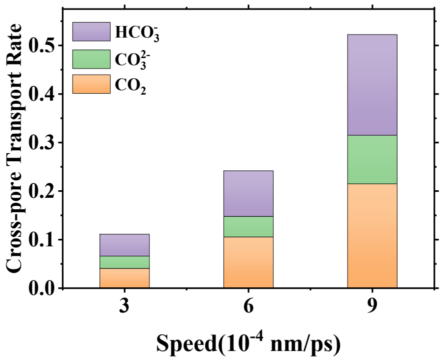

Figure S5. Cross-pore transport rates through the 12.1 Å N-doped micropore at different piston speeds under U=0 V.

To test the robustness of our conclusions to the piston driving speed, simulations of the 12.1 Å N-doped micropore were performed at three piston speeds (3, 6, and 9×10^{-4} nm/ps). As shown in Fig. S5, although the absolute transport rates vary with piston speed, the relative transport ordering among the examined carbon species remains consistent. These results support the robustness of the transport trends obtained under the selected driving condition.

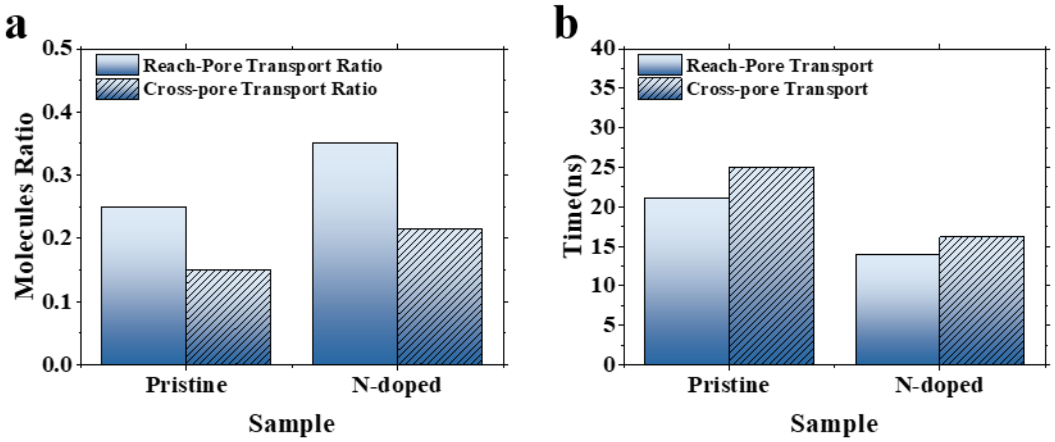

Figure S6. Comparison of CO_2 pore reaching and cross-pore transport. (a) Reach-pore transport ratio and Cross-pore transport ratio for pristine and N-doped TLPs. (b) Pore-mouth residence time of all resolved entry events (Reach-pore transport) and cross-pore times of successfully traversed CO_2 molecules.

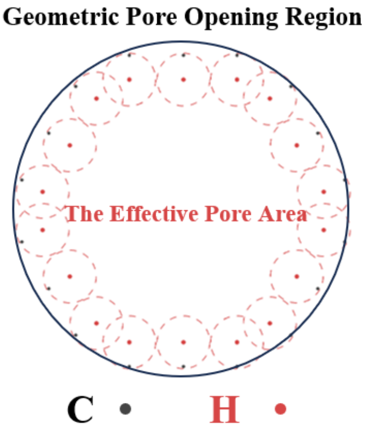

Figure S7. Schematic diagram of the calculation scheme for effective pore area.

The effective pore area A_{eff} is calculated based on the geometric pore opening region by excluding the van der Waals exclusion regions of the pore-edge atoms (Figure S7). The van der Waals radii for the constituent atoms are taken from the commonly used values (r_c=1.70 Å, r_H=1.20 Å，r_N=1.55 Å) . The cross-pore transport rate (TR) is defined as the number of molecules that successfully cross the pore divided by the initial number of the corresponding species in the feed chamber. The area-normalized transport rate is then obtained by dividing TR by A_{eff}.

To elucidate the resistance sources in this system, we decompose the total transport resistance R_{total} of CO_2RR-relevant species traversing multilayer carbon nanocage

micropores into a pore-mouth resistance term R_{mouth} and an intra-channel friction resistance term R_{interior}. Here, R_{mouth} represents the local resistance associated with adsorption/desorption, hydration rearrangement, and molecular entry/release at the pore mouth. Because the pore-mouth environment remains similar for pores with the same diameter, R_{mouth} can be treated as approximately constant with respect to pore depth. R_{interior} represents the cumulative friction resistance during molecular transport through the channel, which can be reasonably assumed to be proportional to pore depth L, i.e., $R_{\text{interior}}(L) = \alpha L$, where α is the internal resistance coefficient per unit depth. Based on this model, we perform a least-squares linear fit of the total resistance data measured at different depths using $R_{\text{total}}(L) = R_{\text{mouth}} + \alpha L$.

References

[1] G. Yang, Y. Kuwahara, K. Mori, C. Louis, H. Yamashita, PdAg alloy nanoparticles encapsulated in N-doped microporous hollow carbon spheres for hydrogenation of CO_2 to formate, Appl. Catal. B Environ. 283 (2021) 119628. https://doi.org/10.1016/j.apcatb.2020.119628.

[2] R. Cui, B. Feng, T. Sun, F. Xu, L. Jiao, Z.-X. Sun, C. Zhou, Y. Yu, L. Yang, X. Wang, H.-Y. Hsu, Q. Wu, Z. Hu, Confining nickel nanoparticles in hierarchical nitrogen-doped carbon nanocages for efficient electrochemical CO_2 reduction to CO, J. Phys. Chem. Lett. (2025) 6305–6312. https://doi.org/10.1021/acs.jpclett.5c00842.

[3] Z. Liu, T. Yan, H. Shi, H. Pan, Y. Cheng, P. Kang, Acidic electrocatalytic CO2 reduction using space-confined nanoreactors, ACS Appl. Mater. Interfaces 14 (2022) 7900–7908. https://doi.org/10.1021/acsami.1c21242.

[4] F. Wang, G. Wang, P. Deng, Y. Chen, J. Li, D. Wu, Z. Wang, C. Wang, Y. Hua, X. Tian, Ultrathin nitrogen-doped carbon encapsulated Ni nanoparticles for highly efficient electrochemical CO_2 reduction and aqueous Zn-CO_2 batteries, Small 19 (2023) 2301128. https://doi.org/10.1002/smll.202301128.

[5] Z. Cao, P. Su, X. Wang, X. Liu, Y. Ma, C. Li, S. Ping Jiang, J. Liu, The structure–activity correlation of single-site Ni catalysts dispersed onto porous carbon spheres toward electrochemical CO2 reduction, Fuel 321 (2022) 124043. https://doi.org/10.1016/j.fuel.2022.124043.
